# Gingival cell growth with antiresorptive treatment combined with corticosteroids or antiestrogen

**DOI:** 10.1002/cre2.382

**Published:** 2021-01-14

**Authors:** Heidi M. Ekholm, Eliisa Löyttyniemi, Tero Soukka, Jaana Rautava

**Affiliations:** ^1^ Department of Oral Pathology and Oral Radiology Institute of Dentistry, University of Turku Turku Finland; ^2^ Department of Clinical Medicine Faculty of Medicine, University of Turku Turku Finland; ^3^ Department of Oral Diseases Turku University Hospital Turku Finland; ^4^ Department of Oral and Maxillofacial Diseases Clinicum, Faculty of Medicine, University of Helsinki and Helsinki University Hospital Helsinki Finland

**Keywords:** antiestrogen, bisphosphonate, corticosteroid, denosumab, oral mucosa

## Abstract

**Objectives:**

Antiresorptive treatment has been shown to impair mucosal cell proliferation, migration, and viability. However, in the clinic, antiresorptives are often used in combination with other drugs. We studied the effect of antiresorptives combined with a corticosteroid or antiestrogen on oral mucosal keratinocytes and fibroblasts.

**Material and methods:**

Human gingival keratinocyte and fibroblast cell lines were exposed to bisphosphonates (BPs) and denosumab in different concentrations and durations together with an antiestrogen or corticosteroid. Changes in cell viability, proliferation and migration after exposures were measured. Data were evaluated with hierarchical linear mixed model for repeated measurements.

**Results:**

Bisphosphonate exposure suppressed keratinocyte and fibroblast cell viability, proliferation, and migration in a time‐dependent manner. Combining a corticosteroid or antiestrogen with BPs further increased this negative effect. Denosumab alone had a mild positive effect on keratinocyte and fibroblast growth. When denosumab was combined with a corticosteroid or antiestrogen, cell growth was suppressed.

**Conclusions:**

Our results show that coexisting medications may increase the negative impact of BPs or denosumab on oral mucosal cells.

## 1 INTRODUCTION

Antiresorptive medications, including bisphosphonates (BPs) and denosumab, are widely used to treat common diseases such as osteoporosis and cancer. BPs have been shown to impair mucosal cell proliferation, migration, and viability in 12 studies (Acil et al., 2012; Basso et al., 2013; Jung et al., 2018; Kim et al., 2011; Landesberg et al., 2008; Manzano‐Moreno et al., 2019; McLeod, Moutasim, Brennan, Thomas, & Jenei, 2014; Pabst et al., 2012; Ravosa, Ning, Liu, et al., 2011; Soydan et al., 2015; Taniguchi et al., 2019; Walter, Pabst, Ziebart, Klein, & Al‐Nawas, 2011). BPs are known to also have a direct toxic effect in vivo on mucosal cells at high concentrations (Otto et al., 2010; Pabst et al., 2012). BPs suppress the biosynthesis of geranylgeraniol pyrophosphate, which is necessary for basic intracellular signaling processes (Hagelauer, Ziebart, Pabst, & Walter, 2015) and ultimately cell growth (Stout, Asiimwe, Birkenstamm, Kim, & Campbell, 2014). Denosumab is a monoclonal antibody that prevents receptor activator of nuclear factor κB ligand (RANKL) from binding to its receptor (RANK), inhibiting osteoclast function (Baron, Ferrari, & Russell, 2011). Besides binding the mineral component of bone and interfering with the action of osteoclasts, RANKL/Osteoprotegerin (OPG) is expressed in epithelial and fibroblast cells (Fujihara et al., 2014; Giannopoulou, Martinelli‐Klay, & Lombardi, 2012; Usui et al., 2016; Vidal et al., 2004). The effect of denosumab on gingival mucosa has been examined in an experimental mouse study, where no changes were detected in fibroblasts (Kuroshima, Al‐Salihi, & Yamashita, 2016).

Medication‐related osteonecrosis of the jaw (MRONJ) is a potential complication of BP/denosumab therapy and a growing problem (Ruggiero et al., 2014; Schiodt et al., 2015). However, MRONJ is a combination of osteonecrosis and ulceration of the mucosa. In order to cure osteonecrosis, keratinocytes and fibroblasts need to close the wound.

The underlying mechanism of MRONJ remains unclear, but it is believed to result from a combination of local and systemic factors (Chiu, Chiang, Chuang, & Chang, 2010; de Boissieu, Gaboriau, Morel, & Trenque, 2016; McGowan, McGowan & Ivanovksi, McGowan, McGowan, & Ivanovski, 2018; Oteri et al., 2018; Ruggiero et al., 2014; Vaszilko et al., 2014). Previous or current corticosteroid therapy is considered a comorbid factor for MRONJ based on epidemiological evidence (McGowan et al., 2018; Ruggiero et al., 2014; Otto et al., 2012). Also, antiestrogen therapy has been found to be more common among MRONJ patients (de Boissieu et al., 2016; Vaszilko et al., 2014). Whether corticosteroid and antiestrogen therapies are in fact causative factors is unknown (Otto, Pautke, Van den Wyngaert, Niepel, & Schiodt, 2018). Both corticosteroids and antiestrogen affect the RANKL/OPG system (Baron et al., 2011; Komori, 2016).

We evaluated the effect of antiresorptive treatment combined with corticosteroid or antiestrogen exposure on oral mucosal keratinocytes and fibroblasts. Based on previous epidemiological and in vitro studies, we hypothesized that BP/denosumab treatment of gingival mucosal cell lines will result in impaired growth and that antiestrogen or corticosteroid therapy will further affect cell growth. The purpose of this study was to show the additive causative effect of corticosteroids and antiestrogen on mucosal wound healing.

## 2 MATERIAL AND METHODS

### 2.1 Cell culture

Spontaneously immortalized human gingival keratinocytes (HMK cell line) (Willberg et al., 2007) of passage 24 and human normal gingival fibroblasts (Ruutu, Rautava, Turunen, Tirri, & Syrjanen, 2017) of passage 8 were cultured in standard 96‐well plates in a humidified incubator with 5% CO_2_ and 95% air at 37°C until the confluence of 60% per well. Cells were passaged at regular intervals using 25% trypsin. Growth media were changed every 3 days using Dulbecco's Modified Eagle's Medium (DMEM) with 1% Penicillin‐Streptomysin‐Neomycin, 1% L‐glutamine, and 10% Fetal Bovine Serum (Thermo Fisher Scientific, Waltham, MA) for fibroblast cells and Keratinocyte‐SFM Medium (Kit) with l‐glutamine, Epidermal Growth Factor and Bovine Pituitary Extract (Thermo Fisher Scientific, Waltham, MA) for HMK cells.

### 2.2 Pharmaceuticals

Two BPs, Zolendronate (Zolendronic acid monohydrate, Sigma‐Aldrich Finland Oy, Finland) and Pamidronate (Pamidronatdinatrium, Hospira UK Limited, Warwickshire, United Kingdom), were used in gradient concentrations of 0, 5, 100, and 500 μmol/L. The antiresorptive Denosumab (XGEVA, Amgen, Thousand Oaks, CA) was used in gradient concentrations of 0, 6, 25, and 600 μg/mL. These concentrations were chosen to cover a wide range of possibilities since no consensus exists on local tissue levels (Chen et al., 2002; Russell, 2011; Scheper et al., 2009). The co‐medications tested included antiestrogen (Faslodex®) and corticosteroid (Di‐Adreson‐F Aquosum®). The concentrations of antiestrogen (8 ng/mL) and corticosteroid (303 ng/mL) were determined in accordance with reports of drug concentrations in plasma with commonly used dosages (Robertson, Odling‐Smee, Holcombe, Kohlhardt, & Harrison, 2003; Varis, Kivisto, & Neuvonen, 2000).

### 2.3 Assays of viability and proliferation

The cells were first exposed to either of the two BPs or denosumab for 24, 48, and 72 h with each concentration and for denosumab additionally for 144 h (day 6). These time points were determined from previous studies (Acil et al., 2012; McLeod et al., 2014; Otto et al., 2010; Ravosa et al., 2011; Reid, Bolland, & Grey, 2007; Soydan et al., 2015; Walter et al., 2011). The cells were then further exposed to antiestrogen or corticosteroid. The control conditions included (1) medium alone (negative control), (2) BP or denosumab alone at each concentration, and (3) antiestrogen or corticosteroid alone at each time point.

Cell viability was tested with AlamarBlue® (ThermoFisher, Waltham, MA) at a concentration of 10%. Experimental detection of AlamarBlue dye was made through spectrophotometry (wave length of 570 nm) after an incubation time of 3 h. The cell media were changed at every assay time point. After the AlamarBlue detections, the cells were assayed for proliferation (CellTiter 96® AQ_ueous_ Non‐Radioactive Cell Proliferation Assay, Promega, Madison, WI) according to the manufacturer's protocol. The unit of these spectrophotometric tests was absorbance in accordance with the manufacturer's protocol. Both assays were conducted in duplicate with four separate replicates.

### 2.4 Migration assay

Cells (confluence 100%) exposed to BPs (0, 5, 100 μmol/L) and denosumab (0, 25 μg/mL) with and without corticosteroid were tested with migration assay Incucyte (IncuCyte Zoom Live Cell Analysis System, Essen BioScence, MI) according to the manufacturer's protocol. The cells were monitored for 24 h. The migration process was automatically recorded with IncuCyte Zoom Software and relative wound density data for statistical analysis. The results were analyzed at the time points of 8, 16, and 24 h after “wounding” with relative wound density percentages, as recommended by the manufacturer. The assay was conducted in eight separate replicates.

### 2.5 Statistics

All results were recorded in Microsoft Excel (Microsoft Corp., Washington, DC), with the exception of the wound healing results, which were analyzed with the appropriate IncuCyte software. Analyses were further performed using SAS System, version 9.4 for Windows (SAS Institute Inc., Cary, NC).

In the migration experiment, changes in absorbance and relative wound density were evaluated with hierarchical linear mixed model for repeated measurements. Drug exposure was handled as a between‐factor and time point of measurement (8, 16, 24 h) as a within‐factor. Interaction between drug exposure and time was also included in the model. The same modeling techniques were used to analyze viability and proliferation. Concentration, time, and drug exposure were included in the model. All statistical tests were performed as two‐sided, with the significance level set at 0.05.

### 2.6 Statement of ethical approval

This article does not contain any studies with human participants or animals performed by any of the authors, and therefore, no ethics approval was needed.

### 2.7 Statement of informed consent

This article does not contain any studies with human participants or animals performed by any of the authors, and therefore, no consent was needed.

## 3 RESULTS

### 3.1 Keratinocyte and fibroblast viability

**FIGURE 1:**
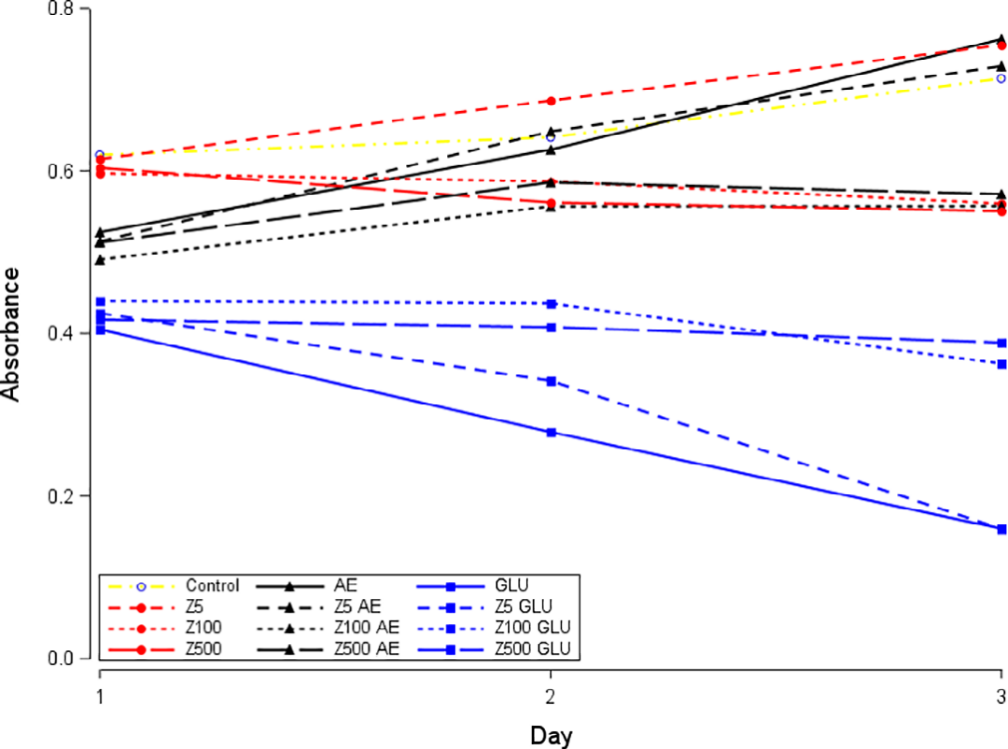
Effects of drugs on epithelial cell viability (absorbance). Zolendronate (Z) at different concentrations (5, 100, 500 μmol/L) is indicated in red. Additive exposures of antiestrogen (AE) or corticosteroid (GLU) are indicated in black and blue, respectively

**FIGURE 2:**
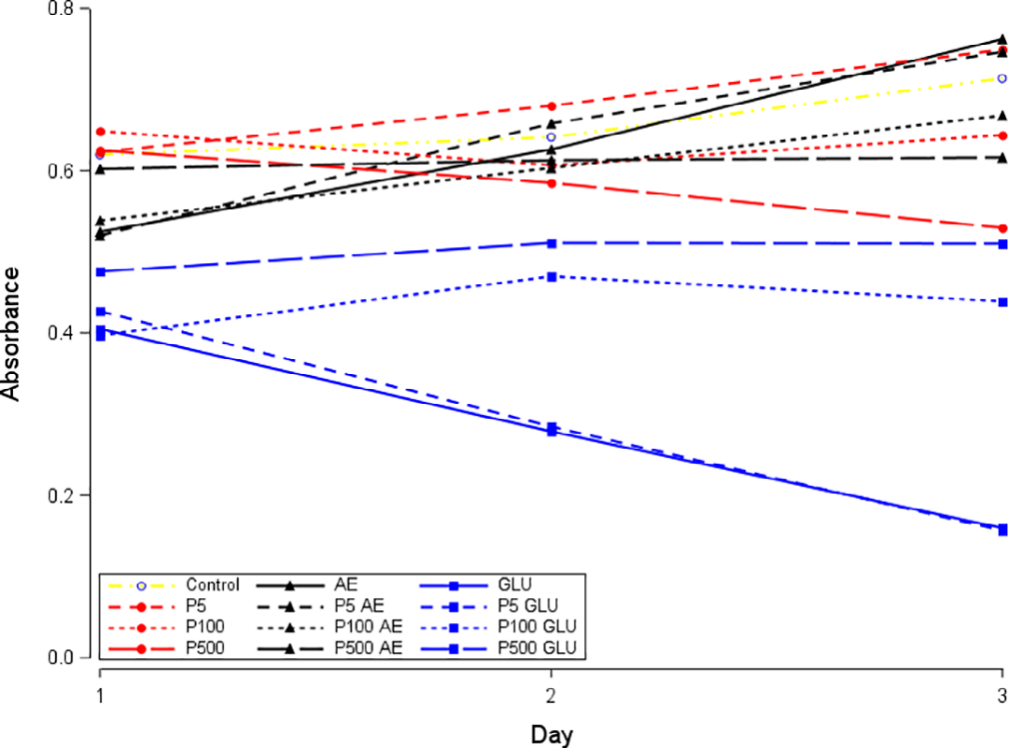
Effects of drugs on epithelial cell viability (absorbance). Pamidronate (P) at different concentrations (5, 100, 500 μmol/L) is indicated in red. Additive exposures of antiestrogen (AE) or corticosteroid (GLU) are indicated in black and blue, respectively

Exposure to both BPs markedly decreased the viability of keratinocytes in a time‐dependent manner (Figure 1). Corticosteroid or antiestrogen exposure alone also resulted in significantly reduced keratinocyte viability (all *p* < .03). Combined exposure to BP and corticosteroid further suppressed viability in keratinocytes, particularly after 24–48 h (all *p* < .001). In a similar fashion, all BP concentrations combined with antiestrogen resulted in reduced keratinocyte viability relative to BP or antiestrogen exposure alone (all *p* < .004) (Table 1, Figures 1 and 2).

**TABLE 1:** Cell growth

Cell type/test	BPs	Denosumab	BPs + glucocorticoid	Denosumab + glucocorticoid	BPs + Antiestrogen	Denosumab + Antiestrogen
Epithelial cell Viability	↓	↑	↓↓	↓	↓↓	↓
Epithelial cell Proliferation	↓	↑	↓↓	↓	↓↓	↓
Epithelial cell Wound healing	↓	↔	↓	↓		
Fibroblast Viability	↓	↑	↓	↓	↓↓	↓
Fibroblast Proliferation	↓	↔	↓	↓	↔	↑
Fibroblast Wound healing	↓	↔	↓	↔		

*Note*: Simplified table of bisphosphonate (BP) and denosumab (D) effects on the gingival epithelial (HMK) and fibroblast cell lines in reference to negative controls.

**FIGURE 3:**
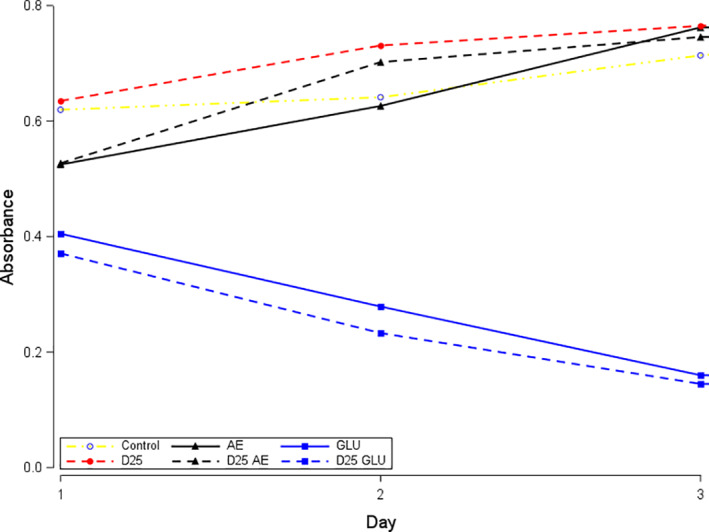
Effects of drugs on epithelial cell viability (absorbance). Denosumab (D) (25 μg/mL) is indicated in red. Additive exposures of antiestrogen (AE) or corticosteroid (GLU) are indicated in black and blue, respectively

Denosumab exposure alone significantly increased the viability of keratinocytes at nearly all of the concentrations and exposure times investigated. However, when denosumab was combined with corticosteroid, a significant decrease in keratinocyte viability was detected compared with denosumab alone (*p* < .0001) (Figure 3). Relative to corticosteroid alone, the viability also further decreased at the higher denosumab concentrations (*p* < .0115). Denosumab combined with antiestrogen also reduced keratinocyte viability 24 h after exposure at all concentrations (all *p* < .004) compared with denosumab exposure alone (*p* < .0082) and compared with antiestrogen alone the result was not significant (*p* > .62).

Both BPs radically decreased the viability of fibroblasts in a time‐dependent manner (*p* < .0001) (Table 1). Corticosteroid exposure alone suppressed fibroblast viability (all *p* < .0001). Decreased viability was detected in fibroblasts exposed to antiestrogen alone 24 h after exposure, but not at later time points. Combined antiestrogen and higher BP concentrations decreased fibroblast viability (all *p* < .0036) compared with either BP or antiestrogen exposure alone. The impact of combined BP and corticosteroid exposure on fibroblast viability was somewhat inconsistent, but always greater than that of BP or corticosteroid exposure alone (all *p* < .0156).

Denosumab exposure resulted in inconsistently increased viability in fibroblasts (Table 1) (*p* < .05). Combined denosumab and corticosteroid exposure decreased fibroblast viability relative to denosumab exposure alone (all *p* < .01). Compared with corticosteroid alone, no significant differences emerged.

### 3.2 Keratinocyte and fibroblast proliferation

Both BPs radically decreased keratinocyte proliferation in a time‐dependent manner (all *p* < .0001) and especially at higher concentrations (100 and 500 μg/mL) (Tables 1 and 2). Corticosteroid exposure alone also suppressed keratinocyte proliferation. Combined BP and corticosteroid exposure of keratinocytes further decreased proliferation after 24–48 h (*p* < .0006) (Table 3). Keratinocytes exposed to antiestrogen alone showed lower proliferation than negative controls at all time points (*p* < .0082). All BP concentrations combined with antiestrogen further decreased proliferation compared with BPs (all *p* < .0001) or antiestrogen alone (all *p* < .04) (Table 4).

**TABLE 2:** Statistically significant proliferation results

Cell type	Drug exposure	Concentration (μmol/L μg/mL)	Differences of LSMeans	Standard error	*p*
Epithelial cells	P	5	0.06421	0.02286	.0063
	P	100	0.2164	0.02286	<.0001
	P	500	0.3265	0.02286	<.0001
	Z	100	0.2837	0.02539	<.0001
	Z	500	0.2842	0.02539	<.0001
	D	600	−0.05623	0.02313	.0167
Fibroblasts	P	100	0.04304	0.01683	.0125
	P	500	0.03312	0.01683	.0526
	Z	5	0.04654	0.01329	.0008
	Z	100	0.09758	0.01329	<.0001
	Z	500	0.07746	0.01329	<.0001
	D	6	−0.04634	0.01772	.0102
	D	25	−0.03816	0.01772	.0336

*Note*: Least Square Means (LSMeans) from bisphosphonates (Pamidronate = P, Zolendronate = Z) or denosumab (D) exposed cells.

**TABLE 3:** Statistically significant proliferation results

Cell type	Drug exposure (μmol/L μg/mL)	Drug exposure (μmol/L μg/mL)	Differences of LSMeans	Standard error	*p*
	VERSUS				
Epithelial cells	Control	GLU	0.1360	0.01998	<.0001
	P(5)	P(5) + GLU	0.08354	0.01387	<.0001
	P(100)	P(100) + GLU	0.05962	0.01387	<.0001
	D(6)	D(6) + GLU	0.1181	0.02208	<.0001
	D(25)	D(25) + GLU	0.1071	0.02208	<.0001
	D(600)	D(600) + GLU	0.1070	0.02208	<.0001
	Z(5)	Z(5) + GLU	0.1434	0.01581	<.0001
	Z(100)	Z(100) + GLU	0.05554	0.01581	.0006
	Z(500)	Z(500) + GLU	0.05908	0.01581	.0003
Fibroblasts	Control	GLU	0.1360	0.01998	<.0001
	P(5)	P(5) + GLU	0.07487	0.01938	.0002
	P(500)	P(500) + GLU	0.07117	0.01938	.0004
	D(6)	D(6) + GLU	0.07272	0.02121	.0008
	D(25)	D(25) + GLU	0.06300	0.02121	.0034
	Z(5)	Z(5) + GLU	0.05792	0.01584	.0004
	Z(500)	Z(500) + GLU	0.04058	0.01584	.0116

*Note*: Comparison of Least Square Means (LSMeans) between bisphosphonates/denosumab exposed cells and bisphosphonate/denosumab with glucocorticoid exposed cells.

Abbreviations: D, denosumab; GLU, glucocorticoid; P, pamidronate; Z, zolendronate.

**TABLE 4:** Statistically significant proliferation results

Cell type	Drug exposure (μmol/L μg/mL)	Drug exposure (μmol/L μg/mL)	Differences of LSMeans	Standard error	*p*
	VERSUS				
Epithelial cells	Control	AE	0.1840	0.01859	<.0001
	P(5)	P(5) + AE	0.1479	0.01267	<.0001
	P(100)	P(100) + AE	0.07504	0.01267	<.0001
	P(500)	P(500) + AE	−0.05712	0.01267	<.0001
	D(6)	D(6) + AE	0.2000	0.01899	<.0001
	D(25)	D(25) + AE	0.1792	0.01899	<.0001
	D(600)	D(600) + AE	0.1883	0.01899	<.0001
	Z(5)	Z(5) + AE	0.1977	0.01473	<.0001
	Z(100)	Z(100) + AE	0.06625	0.01473	<.0001
	Z(500)	Z(500) + AE	0.06221	0.01473	<.0001
Fibroblasts	D(25)	D(25) + AE	−0.05272	0.02226	.0190
	Z(100)	Z(100) + AE	−0.05654	0.01432	.0001

*Note*: Comparison of Least Square Means (LSMeans) between bisphosphonates/denosumab exposed cells and bisphosphonate/denosumab with antiestrogen exposed cells.

Abbreviations: D, denosumab; AE, antiestrogen; P, pamidronate; Z, zolendronate.

Denosumab exposure at the highest concentration increased the proliferation of keratinocytes (*p* < .0167). Combined denosumab and corticosteroid exposure decreased keratinocyte proliferation relative to denosumab alone (*p* < .0001), but not relative to corticosteroid alone. Denosumab combined with antiestrogen reduced keratinocyte proliferation at all time points and concentrations in comparison with denosumab exposure alone (*p* < .0001). Compared with antiestrogen alone, no significant differences emerged (*p* > .5549).

BPs markedly decreased fibroblast proliferation in relation to elapsed time and at all concentrations (all *p* < .0067). Denosumab exposure at lower concentrations increased fibroblast proliferation. Corticosteroid exposure alone also suppressed fibroblast proliferation. Antiestrogen‐exposed fibroblasts had a lower proliferation than negative controls 24 h after exposure (*p* < .0001). The effect of combined corticosteroid and BP exposure on fibroblast proliferation was inconsistent, but always negative relative to BP or corticosteroid exposure alone. The effect of BPs with antiestrogen was inconsistent in fibroblast proliferation.

Denosumab exposure inconsistently increased fibroblast proliferation (*p* < .0336). There was no effect of combined denosumab and corticosteroid exposure on fibroblast proliferation. There was also no effect of combined exposure of denosumab and antiestrogen on fibroblast proliferation (all *p* > .44).

### 3.3 Keratinocyte and fibroblast migration

Keratinocyte migration was decreased after BP exposure. It was 59% lower than in negative controls (*p* < .001) even 72 h after zolendronate (100 μmol/L) exposure. Migration was also decreased with corticosteroid alone. With combined BP and corticosteroid exposure, migration of keratinocytes was further impaired (*p* < .0018) (Table 1).

Migration of only denosumab‐exposed keratinocytes did not differ from negative controls. Combined denosumab‐ and corticosteroid‐exposed keratinocytes showed impaired migration after 8 h, but not at other time points (*p* < .04).

Migration of fibroblasts was impaired after BP exposure. Similarly, it was decreased after corticosteroid alone (all *p* < .001). Fibroblast migration was further impaired with combined BP and corticosteroid exposure (*p* < .0001).

Migration of denosumab‐exposed fibroblasts did not differ from negative controls. Combined exposure of denosumab and corticosteroid yielded no significant differences in migration.

## 4 DISCUSSION

The main finding of this study is that combined treatment of corticosteroids or antiestrogens with BP/denosumab elicits an additive negative impact on oral mucosal cell viability, proliferation, and migration. This effect of combined exposures on oral mucosal cells has not been shown previously in the literature. The finding suggests that oral wound healing may be delayed in patients receiving these treatments.

Viability, proliferation, and migration of oral mucosal cells decreased after BP exposure. This study corroborates previous research, despite some differences in the experimental setup and analysis systems and the cell lines (Acil et al., 2012; Basso et al., 2013; Kim et al., 2011; Landesberg et al., 2008; Manzano‐Moreno et al., 2019; McLeod et al., 2014; Pabst et al., 2012; Ravosa et al., 2011; Soydan et al., 2015; Taniguchi et al., 2019; Walter et al., 2011). In particular, BP concentrations have differed between studies (0.25–500 μmol/L). The high variance in the concentrations of zolendronic acid is due to the slow re‐distribution in bone and the fact that its terminal half‐life has not been adequately determined (Scheper et al., 2009). According to previous studies, the maximum serum concentration (*C*_max_) of zolendronic acid is dose‐dependent, ranging from 403 to 2252 ng/mL (Chen et al., 2002). Some theoretical models (Otto et al., 2010) and a small study of mandibular bone BP concentrations (Scheper et al., 2009) have reported a range of 0.4–126 μmol/L. How high the concentration is in mucosa after increased exposures due to tooth extraction or infection is debatable. BP binds to the bone and during bone resorption it is released into the surrounding tissues in an uncontrolled fashion, resulting in a 100‐fold increase in osseal BP concentrations (Baron et al., 2011; Chen et al., 2002). In our study, BPs had a strong negative effect on gingival epithelial cells in a time‐ and dose‐dependent fashion.

Our results are consistent with those of Kuroshima et al. (2016) who reported that denosumab alone did not impair gingival fibroblast growth. Moreover, we observed that denosumab alone increased mucosal cell growth. Regarding dosages used to treat osteoporosis, denosumab maximum serum concentrations (*C*_max_) of 6 μg/mL occur in 10 days, but in the case of malignancies the dosage is twofold higher and administered two to six times more often (Amgen Ltd, 2017). Based on this, we chose a wide variety of concentrations: 0, 5, 100, and 500 μmol/L for BP and 0, 6, 25, and 600 μg/mL for denosumab, and as the medium was changed the exposure did not continue.

Both corticosteroid and antiestrogen exposure alone impaired epithelial and/or fibroblast cell growth, and the changes were often additive with BP or denosumab. To our knowledge, the in vitro effects of BP/denosumab combined with antiestrogen or corticosteroids have not been previously investigated. It is well known that corticosteroids impair wound healing, suppressing immunological and inflammatory responses (Baxter & Forsham, 1972). Our results demonstrate that exposure of BP‐affected cells to corticosteroids has a more severe negative impact on the cells. We propose that the corticosteroid effect on the RANKL/OPG system (Komori, 2016) together with BP‐induced geranylgeraniol pyrophosphate suppression lead to further suppression of cell growth. This requires confirmation in future studies.

Our findings could offer a preliminary explanation for the previous epidemiological data of corticosteroid and antiestrogen therapy contributing to the risk of MRONJ (de Boissieu et al., 2016; McGowan et al., 2018; Ruggiero et al., 2014). In addition, the results may explain the observation of the prevalence of MRONJ being higher with prolonged BP therapies than with denosumab therapies (de Boissieu et al., 2016; McGowan et al., 2018; Ruggiero et al., 2014). Patients may have delayed wound healing after necrotic bone removal. Denosumab alone did not affect cells negatively, but surprisingly increased cell growth. However, when administered in combination with corticosteroid, denosumab impaired epithelial cell growth. It is therefore possible for denosumab to cause alterations in oral soft tissues in the clinical setting. The frequency of MRONJ caused by zolendronate or denosumab has been reported to be 0.02–6.7% and 0.04–1.9%, respectively (Ruggiero et al., 2014). BP binds to the bone, exposing the oral mucosa to its negative effects over time, while denosumab does not negatively impact the mucosa, nor does the drug have long‐term effects (Ruggiero et al., 2014).

The main strength of this study was that the evaluation of the effects of BP and denosumab on gingival cells was performed using a wide variety of concentrations and measurement time points postexposure and three parallel tests, increasing the reliability of the results. Furthermore, we used gingival cells representing the exact location of MRONJ in the clinical setting. The number of individual cell cultures was high (denosumab *n* = 720, BP *n* = 640, controls *n* = 80), reducing the possibility of significant errors in cell culture.

The major challenge in designing this study was the determination of reagent concentrations, as noted earlier in the discussion. This is due to the uncontrolled release of BPs in excessive bone resorption, therefore causing unpredictable concentrations affecting the oral mucosa. Measuring effects of drug exposures with analysis systems became challenging at the highest BP concentrations since cell confluence decreased radically. A further limitation of the study was the use of monolayer cell cultures. However, monolayer culture is the first step in revealing the phenomenon behind the clinical situation. In any case, further investigations using in vitro 3D‐modeling are needed to map the interactions between cells in MRONJ.

Our results show that corticosteroid and antiestrogen may increase the negative impact of BP or denosumab on oral mucosal cells. This offers one possible explanation for the epidemiological findings that corticosteroid and antiestrogen therapy contribute to the risk of MRONJ.

## CONFLICTS OF INTEREST

The authors declare no conflict of interest.

## AUTHOR CONTRIBUTIONS

Authors TS and JR conceived the study concept. JR and HME developed the theory. HME conducted the experiments with laboratory assistance. EL performed the computations and verified the analytical methods, and HME reported the analytical findings. TS and JR supervised the work. HME drafted the manuscript. All authors discussed the results and contributed to the final manuscript.

## Data Availability

All data related to this manuscript is available upon request.
